# Prevalence and Risk Factors of Anxiety and Depression in Rosacea Patients: A Cross-Sectional Study in China

**DOI:** 10.3389/fpsyt.2021.659171

**Published:** 2021-06-16

**Authors:** Mengting Chen, Zhili Deng, Yingxue Huang, Ji Li

**Affiliations:** ^1^Department of Dermatology, Xiangya Hospital, Central South University, Changsha, China; ^2^National Clinical Research Center for Geriatric Disorders, Xiangya Hospital, Central South University, Changsha, China; ^3^Key Laboratory of Organ Injury, Aging and Regenerative Medicine of Hunan Province, Changsha, China; ^4^Key Laboratary of Aging Biology of Hunan Province, Xiangya Hospital, Central South University, Changsha, China; ^5^Department of Dermatology, The Second Affiliated Hospital of Xinjiang Medical University, Urumqi, China

**Keywords:** depression, anxiety, disease burden, rosacea, epidemiology

## Abstract

Rosacea is a chronic inflammatory skin disease characterized by facial redness and bothersome symptoms. It can exert significant psychological effects and impair the quality of life of patients. To investigate the prevalence and risk predictors of anxiety and depression in rosacea patients, we conducted a cross-sectional study in an outpatient setting. Consecutive patients completed a questionnaire, which included questions on sociodemographic information and severity of signs and symptoms; they also completed the Patient Health Questionnaire and the Generalized Anxiety Disorder scale. Disease burden was assessed using Dermatology Life Quality Index (DLQI), Willing-to-Pay, and Time trade-off. Multivariate analysis was conducted to determine the risk factors for anxiety and depression. A total of 774 patients completed the survey. The prevalence of anxiety was 53.9% (95% CI: 50.4–57.4%) and that of depression was 58.1% (95% CI: 54.7–61.6%). The factors associated with anxiety were age, gender, the need to make appearances at work, severity of self-reported symptoms, the number of rosacea signs and adaptive behaviors, and disease burden. Depression was associated with younger age, more severe self-reported symptoms, more adaptive behaviors, and higher disease burden. After adjusting for demographics, the risk of anxiety or depression increased in young patients who had severe self-reported symptoms, high DLQI scores, and many adaptive behaviors. Taken together, there is a high prevalence of anxiety and depression among Chinese rosacea patients. Younger rosacea patients who have more severe self-reported symptoms and higher disease burden are prone to anxiety and depression.

## Introduction

Rosacea is a chronic inflammatory cutaneous disease with an estimated worldwide prevalence of 5.5% (1). In China, the prevalence of rosacea is up to 3.48% (2). Rosacea mostly affects facial areas and is characterized by recurrent flushing, telangiectasia, persistent erythema, phymas, papules, pustules, edema, and irritated eyes (3, 4). In addition to visible signs, patients with rosacea often complain of high levels of discomfort due to the accompanying subjective symptoms, indicating a possible adverse impact on the psychological well-being of the patients. The pathogenesis of rosacea is complex and multifactorial (5); however, it is widely accepted that numerous exogenous factors, including sun exposure, heat, stress, spicy food, and hot beverages, can initiate rosacea (6). In a high proportion of patients, rosacea is triggered or exacerbated by emotional stress, ranking only behind well-known factors such as sun exposure or elevated temperature (7).

Rosacea patients indeed often experience various symptoms of emotional stress, including depression, anxiety, social phobia, low self-esteem, and frustration (8–11). This might result from that rosacea often involves the central parts of the face and affects the patient's appearance; naturally, the patient will likely be constantly concerned with his/her appearance and how it may be misinterpreted by other people. In addition, the recurrent flushing and the accompanying symptoms are so annoying that they negatively influence the patient's emotional health (12). Besides, the current therapies for rosacea are still limited and transiently effective. This decreases the patient's satisfaction and confidence regarding the treatment, and may further aggravate the patient's psychological distress (13). In general, rosacea and psychological distress are intertwined, thus forming a vicious cycle (14, 15).

Rosacea not only has severe physical and mental health effects, but also imposes a heavy burden on the patients. For the assessment of skin diseases, clinicians commonly use measures/tools, such as the Dermatology Life Quality Index (DLQI) (16, 17), Willingness To Pay (WTP) (18), and Time Trade-Off (TTO) (19), to assess the disease burden. To date, research on rosacea has elucidated the effect of rosacea on the quality of life (20) and its economic (21), and psychological burden (22) on affected patients; however, investigations on anxiety and depression and their association with the disease burden of rosacea are limited. Therefore, to improve the management of rosacea, it is important to comprehensively investigate the psychological status of rosacea patients, especially in China.

In this study, we aimed to investigate the prevalence of anxiety and depression among rosacea patients and to identify the possible demographic/clinical characteristics associated with anxiety and depression in rosacea patients. Identification of the predictors for anxiety/depression in rosacea patients may be valuable to clinicians in daily practice.

## Patients and Methods

### Study Design and Patients

This was a cross-sectional, single center study of 774 rosacea patients conducted at the dermatology outpatient department of Xiangya Hospital of Central South University from May 2018 to August 2019. The inclusion criteria included: (1) patient who visited the hospital for the first time and was independently diagnosed with rosacea by two board-certified dermatologists according to the diagnostic criteria proposed by the National Rosacea Society Expert Committee (3); (2) provision of written informed consent. The exclusion criteria were as follows: (1) patient diagnosed with other concurrent facial skin diseases, such as acne, eczema, systemic lupus erythematosus, seborrheic dermatitis; (2) patient diagnosed with mental illness prior to the occurrence of rosacea; (3) patients who undergo any kinds of trauma that may cause mental illness; (4) patient who had been receiving treatments for rosacea; (5) patient aged <18 years. The study was approved by the ethics committee of the hospital.

### Data Collection

The survey consisted of two parts: a self-administered questionnaire and a face-to-face interview. The self-administered questionnaire included questions on patients' demographic information, patients' self-reported signs and symptoms, symptoms of anxiety/depression, and disease burden-related measures (WTP, TTO, and DLQI), whereas the face-to-face interview included questions on clinical information. The severity of rosacea symptoms was assessed by patients themselves and clinicians using a Visual Analog Scale (VAS) (23)–a horizontal 10 cm VAS with the starting point labeled “none” and the endpoint labeled “very severe” (range of values, 0–10).

We evaluated anxiety and depression separately using a seven-item Generalized Anxiety Disorder scale (GAD-7) and a nine-item Patient Health Questionnaire (PHQ-9) (24, 25). The reliability and validity of the Chinese versions of these scales have been confirmed previously (26). Based on the Diagnostic and Statistical Manual of Mental Disorders-IV (DSM-IV) criteria, the GAD-7 scale focuses on the presence of 7 core anxiety symptoms in the last 2 weeks, and can be categorized into four severity groups: none (0–4), mild (5–9), moderate (10–14), and severe (15–21). Similarly, the total score of PHQ-9 ranges from 0 to 27, and can be categorized into 4 categories: none (0~4), mild (5~9), moderate (10~14), severe (15~19), and very severe (≥19). In the present study, patients who scored 5–9 and ≥10 on the GAD-7 were deemed to have mild and moderate-to-severe anxiety, respectively. Patients who scored 5–9 on the PHQ-9 were classified as having mild depression, whereas those who scored≥10 were considered to have moderate-to-severe depression.

#### Assessment of Disease Burden

1. Quality of life: We utilized the DLQI to assess the effect of rosacea on the quality of life of the patients. The DLQI scores range from 0 to 30, with grade 1 (0~1) indicating no e?ect on the patient's quality of life, grade 2 (2~5) indicating a mild effect, grade 3 (6~10) indicating a moderate effect, grade 4 (11~20) indicating a severe effect, and grade 5 (21~30) indicating very severe impact on the patient's life.
2. Economic burden: We used WTP, which has been widely used in previous studies, to measure the economic burden of rosacea on the patients (18, 21, 27). Patients were asked what percentage and absolute sum of their monthly income they are willing to pay for the disease to be cured.
3. Psychological burden: To further assess the psychological burden of rosacea on the patients, we used TTO as previously described (27). We asked the patients the amount of time they were willing to “trade off” in exchange for a health status without rosacea.

As described previously (27), a definition of “high burden (HB)” includes of the three domains mentioned above, together with the number of adaptive behaviors. Each domain is recognized as positive if its individual value exceeds the median score of all the patients.

### Statistical Analysis

Categorical variables were described as absolute and relative frequencies, whereas continuous variables were expressed as means, standard deviations, and medians. The chi-square test was used to compare the prevalence of anxiety and depression among patients with different sociodemographic and clinical characteristics. The chi-square test and Spearman's correlation analysis were used to assess the relationship between anxiety and depression and disease burden. Variables that were identified as significant using univariate analysis were further investigated using multivariate analysis. Multivariate analysis was performed by using multiple logistic regression (forward elimination: Wald) to identify variables that were independently associated with anxiety and depression among rosacea patients. Relative risk estimates were calculated as odds ratios (ORs) with 95% confidence intervals (CIs). A two-tailed 5% significance level was used in this study. All analyses were performed using SPSS for Windows (version 20.0; SPSS, Chicago, IL, USA).

## Results

### Prevalence of Anxiety and Depression Among Rosacea Patients

A total of 774 patients were enrolled in this study; 417 (53.9%, 95% CI: 50.4–57.4%) suffered from anxiety, whereas 450 (58.1%, 95% CI: 54.7–61.6%) had depression. Among the patients who had anxiety, 20.0% (95% CI: 17.2–22.9%) reported moderate to severe symptoms. For the patients who had depression, 25.8% (95% CI: 22.8–28.9%) reported moderate to severe symptoms.

### Characteristics of Anxiety and Depression Among Rosacea Patients

#### Demographic and Clinical Data

The demographic and clinical information of the participants are summarized in Table 1. Out of the 774 patients enrolled in this study, 75 were males and 699 were females. The mean age of the participants was 33.01 ± 11.13 years and their age range was 18–70 years. Regarding disease duration, 77.6% (*n* = 601) of the patients have had rosacea for less than a year. Furthermore, 29.1% of the patients (*n* = 225) had more than six adaptive behaviors (consciously avoiding heat, sun exposure, and exercise and so on).

**Table 1:** Demographic and clinical factors associated with anxiety or depression.

	**Anxiety**		***p***	**Depression**		***p***	**All (*n =* 774)**
	**Yes (*n =* 417)**	**No (*n =* 357)**		**Yes (*n =* 450)**	**No (*n =* 324)**		
**Age (years)**, ***n*** **(%)**							
Below 20	55 (13.2)	35 (9.8)	0.019	59 (13.1)	31 (9.6)	0.016	90 (11.6)
21–30	172 (41.2)	130 (36.4)		188 (41.8)	114 (35.2)		302 (39.0)
31–40	98 (23.5)	75 (21.0)		101 (22.4)	72 (22.2)		173 (22.4)
41–50	70 (16.8)	87 (24.4)		79 (17.6)	78 (24.1)		157 (20.3)
51 and above	22 (5.3)	30 (8.4)		23 (5.1)	29 (9.0)		52 (6.7)
**Gender**, ***n*** **(%)**							
Male	32 (7.7)	43 (12.0)	0.04	38 (8.4)	37 (11.4)	0.167	75 (9.7)
Female	385 (92.3)	314 (88.0)		412 (91.6)	287 (88.6)		699 (90.3)
**Years of education**, ***n*** **(%)**							
≤12 years	133 (31.9)	136 (38.1)	0.071	148 (32.9)	121 (37.3)	0.199	269 (34.8)
>12years	284 (68.1)	221 (61.9)		302 (67.1)	203 (62.7)		505 (65.2)
**Marital status**, ***n*** **(%)**							
Unmarried	181 (43.4)	135 (37.8)	0.115	199 (44.2)	117 (36.1)	0.024	316 (40.8)
Married	236 (56.6)	222 (62.2)		251 (55.8)	207 (63.9)		458 (59.2)
**Annual household income (**¥**CNY)**, ***n*** **(%)**							
Below 49,999	106 (25.4)	95 (26.6)	0.931	114 (25.3)	87 (26.9)	0.734	201 (26.0)
50,000–99,999	204 (48.9)	172 (48.2)		224 (49.8)	152 (46.9)		376 (48.6)
100,000 and above	107 (25.7)	90 (25.2)		112 (24.9)	85 (26.2)		197 (25.5)
**Appearance requirement for occupation**, ***n*** **(%)**							
None	236 (56.6)	228 (63.9)	0.037	259 (57.6)	205 (63.3)	0.073	464 (59.9)
Low requirement	145 (34.8)	112 (31.4)		153 (34)	104 (32.1)		257 (33.2)
High requirement	36 (8.6)	17 (4.8)		38 (8.4)	15 (4.6)		53 (6.8)
**Duration (month)**, ***n*** **(%)**
Below 12	319 (76.5)	282 (79.0)	0.407	344 (76.4)	257 (79.3)	0.343	601 (77.6)
13 and above	98 (23.5)	75 (21.0)		106 (23.6)	67 (20.7)		173 (22.4)
**Affected areas (amount)**, ***n*** **(%)**							
1	36 (8.6)	28 (7.8)	0.333	38 (8.4)	26 (8.0)	0.495	64 (8.3)
2	65 (15.6)	39 (10.9)		67 (14.9)	37 (11.4)		104 (13.4)
3	95 (22.8)	89 (22.4)		103 (22.9)	72 (22.2)		175 (22.6)
4	95 (22.8)	94 (26.3)		111 (24.7)	78 (24.1)		189 (24.4)
≥5	126 (30.2)	116 (32.5)		131 (29.1)	111 (34.3)		242 (31.3)
**Rosacea signs (amount)**, ***n*** **(%)**							
≤3	23 (5.5)	47 (13.2)	0.003	31 (6.9)	39 (12.0)	0.081	70 (9.0)
4	163 (39.1)	124 (34.7)		176 (39.1)	111 (34.3)		287 (37.1)
5	189 (45.3)	153 (42.9)		199 (44.2)	143 (44.1)		342 (44.2)
6	42 (10.1)	33 (9.2)		44 (9.8)	31 (9.6)		75 (9.7)
**Ocular involvement**, ***n*** **(%)**							
Absent	407 (97.6)	351 (98.3)	0.484	439 (97.6)	319 (98.5)	0.385	758 (97.9)
Present	10 (2.4)	6 (1.7)		11 (2.4)	5 (1.5)		16 (2.1)
**Adaptive behaviors (amount)**, ***n*** **(%)**							
<6	265 (63.5)	284 (79.6)	0.000	293 (65.1)	256 (79.0)	0.000	549 (70.9)
≥6	152 (36.5)	73 (20.4)		157 (34.9)	68 (21.0%)		225 (29.1)

Factors associated with anxiety were younger age, female gender, and higher requirement for appearances in the workplace. Factors linked to depression were younger age and an unmarried status. Moreover, we found that patients who had more rosacea signs displayed a significant association with anxiety, but no association with depression. It is widely accepted that patients have identified many factors that can easily induce or exacerbate rosacea, thus making them consciously avoid these triggers in daily life, named “adaptive behaviors.” Patients who had anxiety or depression tended to have more adaptive behaviors to avoid recurrence and exacerbation of rosacea.

Additionally, we found that the patients' self-reported signs and symptoms, rather than the clinically evaluated ones, were significantly associated with both anxiety and depression in a logistic regression model before and after adjusting for demographics. As shown in Table 2, the number of times flushing occurred in the past 24 h, severity of flushing, flushing-induced sleep disorders, and distress in daily life, burning, stinging, itching, and erythema have significantly positive associations with anxiety and depression. Furthermore, we found that the severity of anxiety or depression increased with increasing VAS scores of self-reported symptoms (Table 3).

**Table 2:** Self-reported and clinician-evaluated signs and symptoms associated with anxiety or depression (demographic data adjusted).

	**Anxiety**		**Depression**	
	**Crude OR (95%CI)**	**Adjusted OR (95%CI)**	**Crude OR (95%CI)**	**Adjusted OR (95%CI)**
**Self-reported signs and symptoms**				
Flush duration	0.995 (0.809–1.223)	1.011 (0.820–1.247)	0.882 (0.716–1.086)	0.891 (0.721–1.100)
Flush times in the past 24 h	1.110^*^ (1.003–1.229)	1.122^*^ (1.011–1.245)	1.213^*^ (1.091–1.349)	1.223^**^ (1.098–1.362)
Flush frequency	0.959 (0.830–1.109)	0.959 (0.826–1.113)	0.980 (0.846–1.134)	0.976 (0.840–1.134)
Severity of flushing	1.634^**^ (1.377–1.940)	1.639^**^ (1.375–1.954)	1.694^**^ (1.423–2.017)	1.722^**^ (1.439–2.060)
Flushing–induced sleep disorders	1.736^**^ (1.460–2.064)	1.817^**^ (1.516–2.178)	1.946^**^ (1.616–2.344)	2.057^**^ (1.693–2.499)
Flushing–induced life distress	1.908^**^ (1.608–2.265)	1.892^**^ (1.589–2.252)	1.892^**^ (1.595–2.246)	1.909^**^ (1.601–2.275)
Severity of burning	1.539^**^ (1.327–1.786)	1.550^**^ (1.329–1.807)	1.545^**^ (1.330–1.796)	1.576^**^ (1.349–1.841)
Severity of stinging	1.565^**^ (1.347–1.817)	1.585^**^ (1.359–1.849)	1.603^**^ (1.375–1.868)	1.639^**^ (1.399–1.919)
Severity of itching	1.429^**^ (1.235–1.653)	1.450^**^ (1.248–1.685)	1.494^**^ (1.287–1.735)	1.539^**^ (1.318–1.796)
Severity of erythema	1.211^*^ (1.031–1.423)	1.241^*^ (1.051–1.465)	1.212^*^ (1.031–1.425)	1.249^**^ (1.057–1.477)
**Clinicians-evaluated signs and symptoms**				
Severity of erythema	0.956 (0.807–1.132)	0.977 (0.820–1.165)	0.860 (0.724–1.021)	0.878 (0.736–1.049)
Severity of papulopustule	0.900 (0.794–1.019)	0.884 (0.778–1.004)	0.889 (0.784–1.008)	0.866^*^ (0.761–0.985)
Severity of phymatous	0.914 (0.758–1.103)	0.927 (0.765–1.123)	0.963 (0.796–1.164)	0.982 (0.808–1.192)
Severity of telangiectasis	1.201 (0.974–1.479)	1.297^*^ (1.044–1.611)	1.134 (0.918–1.400)	1.213 (0.975–1.509)
Severity of plaque	1.250 (0.906–1.724)	1.286 (0.925–1.786)	1.162 (0.841–1.605)	1.197 (0.860–1.666)
Severity of swell	1.377^*^ (1.063–1.785)	1.507^**^ (1.148–1.978)	1.187 (0.921–1.532)	1.300 (0.996–1.696)

* *p < 0.05;*

** *p < 0.01; CI, confidence interval; OR, odds ratio*.

**Table 3:** VAS scores (mean ± SD) of self-reported symptoms in anxiety and depression.

	**Flushing**		**Flushing-induced sleep disorders**		**Flushing-induced life distress**		**Burning**		**Stinging**		**Itching**		**Erythema**	
	**VAS score**	***p***	**VAS score**	***p***	**VAS score**	***p***	**VAS score**	***p***	**VAS score**	***p***	**VAS score**	***p***	**VAS score**	***p***
**Anxiety**														
None	5.21 ± 2.507	0.000	2.07 ± 2.640	0.000	5.18 ± 2.993	0.000	5.09 ± 2.519	0.000	3.24 ± 2.747	0.000	3.82 ± 2.868	0.000	5.44 ± 2.834	0.010
Mild	6.09 ± 2.548		3.21 ± 2.838		6.57 ± 2.708		6.14 ± 2.490		4.61 ± 2.668		4.97 ± 2.705		5.72 ± 2.902	
Moderate	6.88 ± 2.298		3.95 ± 3.421		7.47 ± 2.306		6.36 ± 2.784		4.91 ± 2.536		5.16 ± 2.623		5.72 ± 2.951	
Severe	7.75 ± 2.145		5.15 ± 3.317		8.55 ± 1.880		7.98 ± 2.247		6.35 ± 3.134		6.43 ± 2.791		7.65 ± 2.338	
**Depression**														
None	5.06 ± 2.499	0.000	1.84 ± 2.522	0.000	5.12 ± 3.041	0.000	4.96 ± 2.566	0.000	3.14 ± 2.674	0.000	3.69 ± 2.812	0.000	5.44 ± 2.811	0.001
Mild	6.32 ± 2.508		3.35 ± 2.990		6.55 ± 2.688		6.23 ± 2.442		4.40 ± 2.827		4.96 ± 2.789		5.72 ± 2.953	
Moderate	6.43 ± 2.623		3.84 ± 2.983		7.04 ± 2.679		6.40 ± 2.786		5.24 ± 2.456		5.01 ± 2.786		5.81 ± 2.981	
Severe	6.82 ± 1.980		3.91 ± 3.302		7.64 ± 1.944		6.51 ± 2.437		4.69 ± 2.704		5.64 ± 2.327		5.98 ± 2.641	
Very severe	8.05 ± 2.012		5.85 ± 3.360		8.95 ± 1.468		8.20 ± 1.908		7.90 ± 2.024		7.25 ± 2.425		8.30 ± 2.105	

*VAS, Visual Analog Scale; SD, standard deviation*.

#### Disease Burden

We initially used the DLQI to assess the burden of rosacea. Judging by the DLQI scores, rosacea had a moderate-to-severe impact on the quality of life of 73.9% (*n* = 572) of the patients (DLQI score≥6). The mean ± SD total DLQI score of patients who had anxiety and depression was 14.034 ± 7.511 and 13.336 ± 7.497, respectively, and 8.048 ± 6.348 and 8.407 ± 6.791 for the patients who did not have anxiety and those who did not have depression, respectively. We used WTP and TTO to further evaluate the economic and psychological burden of rosacea on the patients, respectively. Overall, we revealed that the relationship of anxiety/depression with disease burden correlated in a significant way (Table 4).

**Table 4:** Disease burden associated with anxiety or depression.

	**Anxiety**		***p***	**Depression**		***p***	**All (*n =* 774)**
	**Yes (*n =* 417)**	**No (*n =* 357)**		**Yes (*n =* 450)**	**No (*n =* 324)**		
**DLQI**, ***n (%)***							
None	7 (1.7)	53 (14.8)	0.000	13 (2.9)	47 (14.5)	0.000	60 (7.8)
Mild	46 (11.0)	96 (26.9)		60 (13.3)	82 (25.3)		142 (18.3)
Moderate	105 (25.2)	102 (28.6)		109 (24.2)	98 (30.2)		207 (26.7)
Severe	168 (40.5)	88 (24.6)		182 (40.4)	75 (23.1)		257 (33.2)
Very severe	90 (21.6)	18 (5.0)		86 (19.1)	22 (6.8)		108 (14.0)
**Willing to Pay (monthly percent)**, ***n (%), 5 missing***							
0%	44 (10.7)	69 (16.9)	0.000	48 (10.8)	56 (17.3)	0.002	104 (13.5)
0–10%	139 (33.7)	120 (33.7)		143 (32.0)	116 (35.9)		259 (33.7)
10–20%	117 (28.3)	110 (30.9)		133 (29.9)	94 (29.1)		227 (29.5)
20–40%	67 (16.2)	43 (12.1)		72 (16.1)	38 (11.8)		110 (14.3)
>40%	46 (11.1)	23 (6.5)		50 (11.2)	19 (5.9)		69 (9.0)
**Time Tradeoff (month)**, ***n (%)***							
0	214 (51.3)	213 (59.7)	0.001	234 (52.0)	193 (59.6)	0.013	427 (55.2)
0–1	24 (5.8)	35 (9.8)		26 (5.8)	33 (10.2)		59 (7.6)
1–6	31 (7.4)	27 (7.6)		34 (7.6)	24 (7.4)		58 (7.5)
6–12	54 (12.9)	35 (9.8)		60 (13.3)	29 (9.0)		89 (11.5)
12–48	50 (12.0)	31 (8.7)		54 (12.0)	27 (8.3)		81 (10.5)
>48	44 (9.6)	16 (4.5)		42 (9.3)	18 (5.6)		60 (7.8)

*DLQI, Dermatology Life Quality Index*.

#### The Prevalence of Anxiety and Depression Among “High-Burden” Patients

Based on the insights from previous studies, some patients who have rosacea can be defined as a “high-burden (HB)” population who are characterized by an adverse quality of life, adaptive behaviors, and other economic and psychosocial factors (27). Accordingly, applying this definition to our data, we found that HB patients accounted for 17.8% (*n* = 138) of the patients enrolled in this study.

As mentioned previously, we have determined that patients with anxiety or depression had a significantly positive result in three domains that are indicative of HB (DLQI, WTP, and TTO). Further, we also found that there is a positive correlation between the final domain of adaptive behaviors and anxiety (*r* = 0.163, *P* = 0.000) or depression (*r* = 0.169, *P* = 0.000).

### Risk Factors for Anxiety and Depression

Multivariate analysis further identified several predictors for anxiety and depression (Table 5). The risk of anxiety was significantly increased in younger patients, those who experienced more severe stinging, those who had higher DLQI scores, and those who had more adaptive behaviors. Except stinging, the factors mentioned above and more severe flushing-induced life distress predict a higher risk for more severe anxiety. Regarding depression, four factors were significantly associated with an increased risk of depression; they include: younger age; more severe flushing-induced sleep disorders, higher DLQI score, and having more adaptive behaviors. In addition to these factors mentioned, patients who experienced more severe stinging also indicated a higher possibility to suffer from more severe depression.

**Table 5:** Predictors of anxiety or depression identified by multivariate logistic analysis.

	**Anxiety (OR,95%CI)**		**Depression (OR,95%CI)**	
	**Present**	**Moderate-to-severe**	**Present**	**Moderate-to-severe**
Age	0.978 (0.961–0.995)^*^	0.966 (0.942–0.991)^**^	0.969 (0.952–0.986)^**^	0.948 (0.925–0.972)^**^
Stinging	1.354 (1.088–1.685)^**^	ns	ns	1.616 (1.186–2.200)^*^
Flushing-induced sleep disorders	ns	ns	1.602 (1.300–1.973)^**^	1.428 (1.079–1.891)^**^
Flushing-induced life distress	ns	1.559 (1.125–2.162)^**^	ns	ns
DLQI	2.101 (1.715–2.574)^**^	3.012 (2.131–4.258)^**^	1.662 (1.384–1.996)^**^	1.940 (1.482–2.541)^**^
Amount of adaptive behaviors	1.254 (1.094–1.438)^**^	1.412 (1.158–1.722)^**^	1.191 (1.042–1.360)^**^	1.366 (1.139–1.638)^**^

* *p < 0.05;*

** *p < 0.01; CI, confidence interval; OR, odds ratio; ns, no significance*.

## Discussion

In this study, we investigated the prevalence of anxiety and depression in Chinese rosacea patients. In addition, we identified several possible predictive factors for anxiety and depression among rosacea. These results can help clinicians to identify the psychological distress of patients early, thereby facilitating prompt intervention and comprehensive rehabilitation.

Our results showed that 53.9 and 58.1% of the patients had anxiety and depression, respectively, a finding which is comparable to that of a survey conducted by the National Rosacea Society (28). However, the prevalence of anxiety in the present study was much higher than that of a study conducted in Turkey (29). Further, the prevalence of anxiety and depression in the present study was higher than that reported in another Chinese study (30). These discrepancies in the prevalence of anxiety and depression between our data and others' may be attributed to differences in location, types of screening instruments, or the sampling methods used. On the other hand, given that a negative association exists between age and anxiety/depression in our study, the relatively younger age of the patients in the present study may explain the higher prevalence of anxiety and depression recorded in the study.

The causes of the high prevalence of anxiety and depression among rosacea patients are still unclear. However, several plausible reasons may be responsible for the association between anxiety/depression and rosacea. Firstly, rosacea patients tend to experience embarrassment, anxiety, low self-esteem, and decreased quality of life (9, 10, 21) because of their facial face. Secondly, subjective symptoms are always bothersome and may be a source of psychological distress (22). Thirdly, rosacea is extremely susceptible to external stimuli and its severity can fluctuate in accordance with emotional stress; this can lead to recurrence of signs and symptoms, thus further aggravating the patient's psychological sequelae. Finally, psychological distress being involved in the pathogenesis of rosacea (20) may cause an aggravation of inflammation and neurovascular response by increasing the release of cytokines, chemokines, and neurotransmitters from cutaneous cells (31–33).

Our results highlighted many factors that have significant associations with anxiety and depression. From the perspective of demographics, we found that younger patients were more prone to anxiety and depression than older patients. Consistent with the findings of some previous studies (29, 34), we also found that female rosacea patients were more susceptible to anxiety than male patients. However, some other previous studies had contrasting results (9, 22, 35). In the present study, the gender-related differences may be explained by the fact that females are generally more sensitive to changes in their physical appearance than males. Accordingly, we found that patients whose occupations have higher requirements for physical appearance were prone to anxiety. This finding further emphasizes the crucial influence of physical appearance on quality of life, from impairment of social adaptations to psychological sequelae.

Our findings support previous studies that have identified a link between the severity of subjective symptoms and the presence or severity of anxiety/depression in rosacea patients (12). Additionally, we found that the severity of self-reported and clinically evaluated erythema were not parallel. Further, we found that anxiety and depression were more prevalent among patients who had more severe self-reported symptoms but had no significant association with the severity of the symptoms observed by a clinician. However, in daily practice, clinicians often focus exclusively on the visible signs and overlook the invisible symptoms; this makes the patient feel that his/her subjective symptoms are easily neglected.

In addition, we also investigated the possible association between anxiety/depression and the disease burden of rosacea. Consistent with results of prior studies, we found that DLQI score has a significantly positive association with anxiety and depression (22, 30). We also identified a correlation between the self-perceptions of flushing-related sleep disorders/life distress and the presence or severity of anxiety/depression in rosacea patients. These findings highlight that the mental and functional impairments associated with the disease largely contribute to anxiety or depression. In addition to DLQI, we utilized WTP to assess the economic burden of rosacea (18, 21, 26, 36), and TTO to evaluate the psychosocial burden of rosacea (27). We observed that patients who had anxiety and depression preferred to invest more money and trade more of their lifetime for a complete cure of rosacea. In addition to these classic indicators of disease burden, “adaptive behavior” was another challenging issue for the patients. It is widely accepted that patients have identified many factors that can easily induce or exacerbate rosacea, thus making them consciously avoid these triggers in daily life (7, 29); these avoidance habits are termed “adaptive behaviors.” Based on the results, we found that patients who had more adaptive behaviors were more likely to be anxious or depressed, indicating that the more lifestyle habits they had to change, the larger the impact of the disease on their lives, and the higher the psychological burden.

A new concept of HB in rosacea patients was recently proposed. This concept indicates that for HB rosacea patients, at least three of four domains are positive (DLQI, WTP, TTO, and lifestyle) (27, 29). Based on this definition, we identified 17.8% HB rosacea patients in the present study, a finding which is similar to that of a previous study (27). Further, we found that anxiety and depression were more prevalent and severe in HB rosacea patients. Taken together, these results indicate a close relationship between anxiety and depression and the high disease burden of rosacea. The recognition of HB patients may encourage clinicians to screen for anxiety and depression in rosacea patients.

Considering the high incidence of anxiety and depression among rosacea patients, especially in HB patients, it is necessary to identify patients who have anxiety or depression to avoid serious consequences such as suicide. Our results highlighted that younger age, more severe self-reported symptoms, higher DLQI score, and having more adaptive behaviors are predictive factors for the presence and severity of anxiety and depression.

Since utilizing GAD-7 and PHQ-9 scales that have numerous questions is not practical in a clinical setting due to the restricted duration of clinical visits, asking questions using the VAS would be of simple and value. Therefore, to screen for anxiety and depression, we recommend the use of the following questions in clinical practice:

1. Score the effect of rosacea on your sleep using the VAS.
2. Score the effect of rosacea on your daily life using the VAS.
3. Describe the severity of your symptoms (including flushing, stinging, burning, and itching) using the VAS
4. How many lifestyle changes have you made due to rosacea?

These questions will help physicians to recognize patients who have psychological burdens and o?er them psychological counseling earlier.

This study has some limitations. Firstly, as this was a single-center survey, the sample population is not representative of the whole population of China. Secondly, the choice of scale for the assessment of anxiety and depression may have the potential to skew the detection of the presence and severity of anxiety and depression in rosacea patients. Lastly, we overlooked the prevalence of anxiety and depression in rosacea patients after proper treatments, and thus not being able to provide comprehensive data to fully demonstrate that rosacea can cause anxiety and depression. However, these limitations are unlikely to confound the comparison of the groups evaluated in this study. We will continue to explore these issues more thoroughly and comprehensively in further studies.

In conclusion, the proportion of rosacea patients who have anxiety or depression is large. However, the psychological distress experienced by these patients is usually neglected by clinicians. Our study revealed that younger age, more severe self-reported symptoms, and higher disease burden may indicate the possibility of anxiety or depression, especially when clinical signs and self-reported symptoms are inconsistent or if patients have apparently impaired quality of life. Psychological intervention should be taken into consideration when prescribing atreatment regimen.

## Data Availability Statement

The original contributions presented in the study are included in the article/supplementary material, further inquiries can be directed to the corresponding authors.

## Ethics Statement

The studies involving human participants were reviewed and approved by The protocol for this study was established, according to the ethical guidelines of the Helsinki Declaration and approved by the ethical committee of the Xiangya Hospital, Central South University, China (IRB number 201404361). The patients/participants provided their written informed consent to participate in this study.

## Author Contributions

MC and ZD were responsible for writing and revising the article, as well as the experiment design. YH were responsible for the guidance on experiment design and statistical methods. JL were responsible for the participant enrolment and the management and quality control of the research process. All authors gave their scientific contribution and have approved the final manuscript.

## Conflict of Interest

The authors declare that the research was conducted in the absence of any commercial or financial relationships that could be construed as a potential conflict of interest.

## References

[1] Gether L Overgaard LK Egeberg A Thyssen JP. Incidence and prevalence of rosacea: a systematic review and meta-analysis. Br J Dermato. (2018) 179:282–9. 10.1111/bjd.16481 29478264

[2] Li J Wang B Deng Y Shi W Jian D Liu F. Epidemiological features of rosacea in Changsha, China: a population-based, cross-sectional study. J Dermato. (2020) 47:497–502. 10.1111/1346-8138.15301 32207167

[3] Gallo RL Granstein RD Kang S Mannis M Steinhoff M Tan J. Standard classification and pathophysiology of rosacea: the 2017 update by the National Rosacea Society Expert Committee. J Eur Acad Dermatol Venereol. (2018) 78:148–55. 10.1016/j.jaad.2017.08.037 29089180

[4] Thiboutot D Anderson R Cook-Bolden F Draelos Z Gallo RL Granstein RD. standard management options for rosacea: the 2019 update by the national rosacea society expert committee. Eur Acad Dermatol Venereol. (2020) 82:1501–10. 10.1016/j.jaad.2020.01.077 32035944

[5] Buddenkotte J Steinhoff M. Recent advances in understanding and managing rosacea. F1000Research. (2018) 7:F1000. 10.12688/f1000research.16537.1 30631431 PMC6281021

[6] Woo YR Lim JH Cho DH Park HJ. Rosacea: molecular mechanisms and management of a chronic cutaneous inflammatory condition. Int j mol sci. (2016) 17:1562. 10.3390/ijms17091562 27649161 PMC5037831

[7] National Rosacea Society. Rosacea Triggers Survey. (2015). Available online at: https://www.rosacea.org/patients/rosacea-triggers/rosacea-triggers-survey (accessed May 25, 2021).

[8] Cardwell LA Nyckowski T Uwakwe LN Feldman SR. Coping mechanisms and resources for patients suffering from rosacea. Dermatol Clin. (2018) 36:171–4. 10.1016/j.det.2017.11.013 29499801

[9] Halioua B Cribier B Frey M Tan J. Feelings of stigmatization in patients with rosacea. J Eur Acad Dermatol Venereol. (2017) 31:163–8. 10.1111/jdv.13748 27323701

[10] Gupta MA Gupta AK Chen SJ Johnson AM. Comorbidity of rosacea and depression: an analysis of the national ambulatory medical care survey and national hospital ambulatory care survey–outpatient department data collected by the US national center for health statistics from 1995 to 2002. Br J Dermato. (2005) 153:1176–81. 10.1111/j.1365-2133.2005.06895.x 16307654

[11] Oussedik E Bourcier M Tan J. Psychosocial burden and other impacts of rosacea on patients' quality of life. Dermatol Clin. (2018) 36:103–13. 10.1016/j.det.2017.11.005 29499793

[12] Abram K Silm H Maaroos H-I Oona M. Subjective disease perception and symptoms of depression in relation to healthcare-seeking behaviour in patients with rosacea. Acta Dermato-Venereol. (2009) 89:488–91. 10.2340/00015555-0716 19734974

[13] Johnston SA Krasuska M Millings A Lavda AC Thompson AR. Experiences of rosacea and its treatment: an interpretative phenomenological analysis. Br J Dermato. (2018) 178:154–60. 10.1111/bjd.15780 28667759

[14] Dirschka T Micali G Papadopoulos L Tan J Layton A Moore S. Perceptions on the psychological impact of facial erythema associated with rosacea: results of international survey. Dermatol Ther. (2015) 5:117–27. 10.1007/s13555-015-0077-2 26022994 PMC4470961

[15] Beerman H. Are-evaluation of the rosacea complex. Am J Med Sci. (1956) 232:458–73. 10.1097/00000441-195610000-00013 13362258

[16] Huynh TT. Burden of disease: the psychosocial impact of rosacea on a patient's quality of life. Am Health Drug Benefits. (2013) 6:348–54. 24991368 PMC4031723

[17] Finlay AY Khan GK. Dermatology life quality index (DLQI)–a simple practical measure for routine clinical use. Clin exp dermatol. (1994) 19:210–6. 10.1111/j.1365-2230.1994.tb01167.x 8033378

[18] Seidler AM Bayoumi AM Goldstein MK Cruz PD Jr Chen SC. Willingness to pay in dermatology: assessment of the burden of skin diseases. J Invest Dermatol. (2012) 132:1785–90. 10.1038/jid.2012.50 22418874

[19] Schiffner R Schiffner-Rohe J Gerstenhauer M Hofstadter F Landthaler M Stolz W. Willingness to pay and time trade-off: sensitive to changes of quality of life in psoriasis patients? Br J Dermatol. (2003) 148:1153–60. 10.1046/j.1365-2133.2003.05156.x 12828743

[20] Aksoy B Altaykan-Hapa A Egemen D Karagoz F Atakan N. The impact of rosacea on quality of life: effects of demographic and clinical characteristics and various treatment modalities. Br J Dermatol. (2010) 163:719–25. 10.1111/j.1365-2133.2010.09894.x 20545683

[21] Beikert FC Langenbruch AK Radtke MA Augustin M. Willingness to pay and quality of life in patients with rosacea. J Europ Acad Dermatol Venereol. (2013) 27:734–8. 10.1111/j.1468-3083.2012.04549.x 22583164

[22] Boehm D Schwanitz P Gissendanner SS Schmid-Ott G Schulz W. Symptom severity and psychological sequelae in rosacea: results of a survey. Psychol Health Med. (2014) 19:586–91. 10.1080/13548506.2013.841968 24088195

[23] Kroenke K Spitzer RL Williams JBW. The PHQ-9–validity of a brief depression severity measure. J General Internal Med. (2001) 16:606–13. 10.1046/j.1525-1497.2001.016009606.x PMC1495268 11556941

[24] Manea L Gilbody S McMillan D. Optimal cut-off score for diagnosing depression with the patient health questionnaire (PHQ-9): a meta-analysis. Canad Med Assoc J. (2012) 184:E191–6. 10.1503/cmaj.110829 22184363 PMC3281183

[25] Wang H Li T Yuan W Zhang Z Wei J Qiu G. Mental health of patients with adolescent idiopathic scoliosis and their parents in china: a cross-sectional survey. BMC Psychiatry. (2019) 19:147. 10.1186/s12888-019-2128-1 31088538 PMC6515648

[26] Beikert FC Langenbruch AK Radtke MA Kornek T Purwins S Augustin M. Willingness to pay and quality of life in patients with atopic dermatitis. Arch Dermatol Res. (2014) 306:279–86. 10.1007/s00403-013-1402-1 23982630

[27] Tan J Steinhoff M Bewley A Gieler U Rives V. Characterizing high-burden rosacea subjects: a multivariate risk factor analysis from a global survey (vol 18, pg 1, 2019). J Dermatolog Treat. (2020) 31:210. 10.1080/09546634.2019.1665305 31120382

[28] National Rosacea Society. Red Alert: Rosacea Awareness Month Highlights Potential Increased Health Risks. (2016). Available online at: https://www.rosacea.org/press/2016/april/red-alert-rosacea-awareness-month-highlights-potential-increased-health-risks

[29] Uysal PI Akdogan N Hayran Y Oktem A Yalcin B. Rosacea associated with increased risk of generalized anxiety disorder: a case-control study of prevalence and risk of anxiety in patients with rosacea. Anais Brasileiros De Dermatol. (2019) 94:70−9. 10.1016/j.abd.2019.03.002 31789266 PMC6939083

[30] Wu Y Fu C Zhang W Li C Zhang J. The dermatology life quality index (DLQI) and the hospital anxiety and depression (HADS) in Chinese rosacea patients. Psychol Health Med. (2018) 23:369–74. 10.1080/13548506.2017.1361540 28797174

[31] Li J Yuan X Tang Y Wang B Deng Z Huang Y. Hydroxychloroquine is a novel therapeutic approach for rosacea. Int Immunopharmacol. (2020) 79:106178. 10.1016/j.intimp.2019.106178 31918061

[32] Chen M Xie H Chen Z Xu S Wang B Peng Q. Thalidomide ameliorates rosacea-like skin inflammation and suppresses NF-kappa B activation in keratinocytes. Biomed Pharmacother. (2019) 116:109011. 10.1016/j.biopha.2019.109011 31132668

[33] Liu T Deng Z Xie H Chen M Xu S Peng Q. ADAMDEC1 Promotes skin inflammation in rosacea via modulating the polarization of m1 macrophages. Biochem Biophys Res Commun. (2020) 521:64–71. 10.1016/j.bbrc.2019.10.073 31627897

[34] Chen Q Tang Y Shi X Yang X Shan S Wang X. Prevalence, clinical characteristics and health-related quality of life of rosacea in chinese adolescents: a population-based study. J Eur Acad Dermatol Venereol. (2020) 34:e737–9. 10.1111/jdv.16576 32362044

[35] Heisig M Reich A. Psychosocial aspects of rosacea with a focus on anxiety and depression. Clin Cosmetic Invest Dermatol. (2018) 11:103–7. 10.2147/ccid.S126850 29551906 PMC5844253

[36] Radtke MA Schaefer I Gajur A Langenbruch A Augustin M. Willingness-to-pay and quality of life in patients with vitiligo. Br J Dermato. (2009) 161:134–9. 10.1111/j.1365-2133.2009.09091.x 19298268

